# Predictors of pathologic complete response in early-stage triple-negative breast cancer treated with neoadjuvant chemo-immunotherapy: a multi-institution study

**DOI:** 10.1007/s10549-026-07984-8

**Published:** 2026-05-28

**Authors:** Alexis LeVee, Bethania Santos, Megan Wong, Nora Ruel, Daniel Schmolze, Irene Kang, Karen Tsai, Joanne Mortimer, Heather McArthur

**Affiliations:** 1 Division of Hematology and Oncology, University of California Los Angeles, 10833 Le Conte Ave, Los Angeles, CA 90095 USA; 2 Department of Medical Oncology, City of Hope Comprehensive Cancer Center, Duarte, CA 91010 USA; 3 Division of Hematology and Oncology, UT Southwestern Medical Center, Dallas, TX 75235 USA; 4 Department of Biostatistics, City of Hope Comprehensive Cancer Center, Duarte, CA 91010 USA; 5 Department of Pathology, City of Hope Comprehensive Cancer Center, Duarte, CA 91010 USA; 6 Department of Endocrinology, City of Hope Comprehensive Cancer Center, Duarte, CA 91010 USA

**Keywords:** Triple-negative breast cancer, Immunotherapy, Pembrolizumab, Pathologic complete response, Neoadjuvant chemotherapy

## Abstract

**Introduction:**

The KEYNOTE-522 clinical trial demonstrated that the addition of pembrolizumab to 8 cycles of neoadjuvant chemotherapy (NAC) improves pathologic complete response (pCR) rates and overall survival in early-stage triple-negative breast cancer (TNBC). However, predictors of response and the benefit of alternative NAC backbones with immunotherapy are not known. This multi-institutional study evaluates clinical factors and treatment variables associated with pCR following NAC plus pembrolizumab in a diverse, real-world cohort.

**Methods:**

This multi-institution retrospective study analyzed patients with early-stage TNBC diagnosed between July 1, 2021, and December 31, 2023, across three hospital systems. Eligible patients received at least one cycle of NAC and pembrolizumab. Predictors of pCR were assessed using logistic regression.

**Results:**

Of the 374 patients included, the pCR rate was 61.2%. The cohort was racially and ethnically diverse, with 29.1% of patients identifying as non-White. Almost half (48.4%) had a BMI ≥30, and 70 patients (18.7%) had pre-existing diabetes mellitus. Approximately two-thirds (64.4%) completed 8 cycles of neoadjuvant pembrolizumab, while 72.5% completed 8 cycles of NAC. On univariate analysis, age less than 55 years (*p* = 0.03), absence of diabetes mellitus (*p* = 0.03), high tumor grade (*p* = 0.005), the completion of 8 or more cycles of neoadjuvant pembrolizumab (*p* = 0.04) and 8 cycles of NAC (*p* = 0.03) were associated with pCR. There was a trend towards higher pCR rates with the receipt of at least one cycle of anthracycline-cyclophosphamide (*p* = 0.07). Lack of diabetes mellitus (*p* = 0.03) and high tumor grade (*p* = 0.004) remained significant on multivariate analysis.

**Conclusion:**

This study supports the use of KEYNOTE-522 in a diverse, real-world population of patients with early-stage TNBC. Patients with diabetes were less likely to achieve a pCR, suggesting a potential impact of glucose metabolism on chemo-immunotherapy resistance.

## Introduction

 The KEYNOTE-522 trial demonstrated that the addition of pembrolizumab to neoadjuvant chemotherapy (NAC) improves pathologic complete response (pCR) rates and survival outcomes in early-stage triple-negative breast cancer (TNBC) [1, 2]. However, generalizability of this treatment regimen to a real-world, diverse patient population remains uncertain, as only 4.5% of patients in the trial were Black, 11% were age 65 or older, and all patients were required to have adequate organ function and ECOG performance status [3]. As such, determining the clinical efficacy of neoadjuvant chemo-immunotherapy in a diverse patient population is needed.

While studies utilizing biomarkers to predict immune checkpoint inhibitor (ICI) benefit in early-stage TNBC are ongoing, there are currently no companion biomarkers to inform clinical decisions about the use of chemo-immunotherapy in early-stage TNBC. Although higher PD-L1 expression was significantly associated with higher pCR rates in the phase 1b KEYNOTE-173 study, the subsequent KEYNOTE-522 phase 3 study demonstrated benefit with pembrolizumab regardless of PD-L1 status [1, 4]. Other potential predictive biomarkers of ICI efficacy include stromal tumor-infiltrating lymphocytes (sTILs) [4–6], immune gene expression signatures [7, 8], and spatial organization of immune cell infiltrates [9] within the tumor microenvironment, but they are not currently adopted to inform decisions about NAC with pembrolizumab. Thus, identification of predictors of pCR may refine therapeutic recommendations.

In addition to refining patient selection for ICI, questions remain regarding the optimal chemotherapy backbone in combination with ICI. The KEYNOTE-522 trial included four chemotherapy agents in combination with pembrolizumab, specifically four cycles of carboplatin in combination with taxane and four cycles of anthracycline plus cyclophosphamide. However, whether each of these four chemotherapy agents are necessary in combination with ICI is unclear, with ongoing trials evaluating chemotherapy de-escalation strategies to improve clinical outcomes while minimizing toxicity.

This multi-institutional study explored key clinical and treatment factors associated with pCR in patients with early-stage TNBC treated with neoadjuvant chemo-immunotherapy, providing insights on patient selection and optimizing therapeutic strategies.

## Methods

Patients with early-stage TNBC diagnosed between July 1, 2021, and December 31, 2023, were identified from three hospital system databases (City of Hope Comprehensive Medical Center, University of Texas Southwestern, and Parkland Memorial Hospital). This study was approved by the institutional review boards of each institution.

Patients were eligible if they received at least one cycle of NAC and at least one cycle of neoadjuvant pembrolizumab. TNBC was defined as ER and PR expression ≤10% by immunohistochemistry (IHC) and HER2-negative (IHC 0/1 + or negative by FISH) per American Society of Clinical Oncology (ASCO)/College of American Pathologists (CAP) guidelines.

Data collection was performed via manual chart review of the electronic medical record through April 15, 2025, at which point data abstraction was cut off. Demographic information included age at diagnosis, race, ethnicity, sex, and menopausal status. Tumor characteristics included primary tumor and nodal stage, histologic subtype, grade, and Ki-67 expression. Tumor characteristics were obtained from diagnostic imaging and pathology reports of core biopsy specimens. Treatment data including neoadjuvant treatment initiation and end date, type of chemotherapy, and number of cycles were obtained. Pathologic information was assessed from pathology reports of surgical specimens. pCR was defined as ypT0/Tis and ypN0.

Patient and clinical characteristics were compared using two-sample tests (t-test or Wilcoxon rank-sum test for continuous variables and chi-square test for categorical data) between the two groups as defined by pCR status. Univariate and multivariate logistic regression was performed to identify variables associated with pCR including clinical factors, tumor characteristics, and treatment variables.

Event-free survival (EFS) and overall survival (OS) were calculated from the date of diagnosis with EFS defined as time to invasive ipsilateral breast tumor recurrence, local/regional invasive recurrence, distant recurrence, death from breast cancer, death from non-breast cancer, or death from an unknown cause, whichever presented first. Survival probability distribution was calculated using the Kaplan-Meier method and stratified according to pCR status. Patients were censored at the date of last follow-up. A threshold of 0.05 was used to assess the significance of all statistical analyses. All analyses were conducted in SAS9.4.

## Results

### All patients

The study included 374 patients with a median age at diagnosis of 51.6 years (range 20.8–87.7). Most patients were female except for 1 male. The cohort was racially and ethnically diverse, with approximately 29.1% of patients identifying as non-White and 34.5% identifying as Hispanic/Latino. The median body mass index (BMI) was 29.6 kg/m2. Almost one-third of patients had hypertension and hyperlipidemia, and almost one in five patients had diabetes mellitus. Most (94.6%) patients had clinical stage II or III disease. Patient and tumor characteristics are summarized in Table 1 according to pCR status.

Table 2 demonstrates the treatment characteristics according to pCR status. Approximately two-thirds of patients (64.4%) completed 8 cycles of neoadjuvant pembrolizumab, and 72.5% of patients completed 8 cycles of NAC, irrespective of the NAC backbone delivered. The rate of completion of at least 75% of carboplatin and taxane doses as recommended by KEYNOTE-522 was 24.6% and 44.9%, respectively. 75.9% of patients completed 4 cycles of anthracycline-cyclophosphamide.

**Table 1:** Patient and tumor characteristics according to pCR status

	All Patients (*n* = 374)	pCR (*n* = 229)	Non-pCR (*n* = 145)	*P*-value
**Age at diagnosis (years), median (range)**	51.6 (20.8–87.7)	50.4 (20.8–87.7)	54.5 (24.5–84.0)	0.005
**Gender**				
Female	373 (99.7%)	229 (100.0%)	144 (99.3%)	0.2
Male	1 (0.3%)	0 (0.0%)	1 (0.7%)	
**BMI**,** median (kg/m2) (range)**	29.6 (15.3–59.6)	30.0 (16.8–52.9)	29.1 (15.3–59.6)	0.5
**BMI group**				
Not obese (BMI < 30)	193 (51.6%)	113 (49.3%)	80 (55.2%)	0.3
Obese/Morbidly obese (BMI ≥30)	181 (48.4%)	116 (50.7%)	65 (44.8%)	
**Race**				
Asian	43 (11.5%)	23 (10.0%)	20 (13.8%)	0.3
Black or African American	66 (17.6%)	37 (16.2%)	29 (20.0%)	
White	248 (66.3%)	160 (69.9%)	88 (60.7%)	
Other/Unknown	17 (4.5%)	9 (3.9%)	8 (5.5%)	
**Ethnicity**				
Hispanic/Latino	129 (34.5%)	86 (37.6%)	43 (29.7%)	0.3
Non-Hispanic/Latino	236 (63.1%)	137 (59.8%)	99 (68.3%)	
Unknown	9 (2.4%)	6 (2.6%)	3 (2.1%)	
**Comorbidities present**				
Hypertension	121 (32.4%)	67 (29.3%)	54 (37.2%)	0.11
Hyperlipidemia	118 (31.6%)	70 (30.6%)	48 (33.1%)	0.6
Diabetes mellitus	70 (18.7%)	35 (15.3%)	35 (24.1%)	0.03
**Menopausal status**				
Pre-menopausal	171 (45.7%)	110 (48.0%)	86 (59.3%)	0.09
Post-menopausal	196 (52.4%)	115 (50.2%)	56 (38.6%)	
Unknown/Not applicable	7 (1.9%)	4 (1.7%)	3 (2.1%)	
**TNM stage**				
I	20 (5.4%)	12 (5.2%)	8 (5.5%)	1.0
II	161 (43.0%)	100 (43.7%)	61 (42.1%)	
III	193 (51.6%)	117 (51.1%)	76 (52.4%)	
**Clinical tumor stage**				
T0	2 (0.5%)	2 (0.9%)	0 (0.0%)	0.3
T1	42 (11.2%)	23 (10.0%)	19 (13.1%)	
T2	227 (60.7%)	143 (62.4%)	84 (57.9%)	
T3	71 (19.0%)	45 (19.7%)	26 (17.9%)	
T4	30 (8.0%)	14 (6.1%)	16 (11.0%)	
TX	2 (0.5%)	2 (0.9%)	0 (0.0%)	
**Regional node classification**				
N0	189 (50.5%)	117 (51.1%)	72 (49.7%)	1.0
N1	143 (38.2%)	86 (37.6%)	57 (39.3%)	
N2	18 (4.8%)	12 (5.2%)	6 (4.1%)	
N3	20 (5.3%)	12 (5.2%)	8 (5.5%)	
NX	4 (1.1%)	2 (0.9%)	2 (1.4%)	
**Histopathology**				
Ductal	337 (90.1%)	210 (91.7%)	127 (87.6%)	0.4
Lobular	5 (1.3%)	2 (0.9%)	3 (2.1%)	
Unspecified/Other	32 (8.5%)	17 (7.4%)	15 (10.3%)	
**Tumor grade**				
1	1 (0.3%)	0 (0.0%)	1 (0.7%)	0.02
2	49 (13.1%)	21 (9.2%)	28 (19.3%)	
3	318 (85.0%)	205 (89.5%)	113 (77.9%)	
Unknown	6 (1.6%)	3 (1.3%)	3 (2.1%)	
**Ki-67 index (%)**,** median (range)**	68 (3, 100)	80 (5, 100)	68 (3, 98)	< 0.0001
**Estrogen receptor**				
< 1%	320 (85.6%)	196 (85.6%)	124 (85.5%)	1.0
1–10%	54 (14.4%)	33 (14.4%)	21 (14.5%)	
**Progesterone receptor**				
< 1%	348 (93.0%)	215 (93.9%)	133 (91.7%)	0.4
1–10%	25 (6.7%)	14 (6.1%)	11 (7.6%)	
Unknown	1 (0.3%)	0 (0.0%)	1 (0.7%)	
**HER2 IHC**				
0	158 (42.5%)	96 (41.9%)	62 (42.8%)	0.3
1+	105 (28.1%)	69 (30.1%)	36 (24.8%)	
2+	55 (14.7%)	28 (12.2%)	27 (18.6%)	
Unknown	56 (15.0%)	36 (15.7%)	20 (13.8%)	

Abbreviations: BMI, body mass index; IHC, immunohistochemistry

**Table 2:** Neoadjuvant treatment characteristics and clinicopathologic outcomes according to pCR status

	All Patients (*n* = 374)	pCR (*n* = 229)	Non-pCR (*n* = 145)	*P*-value
**Cycles of pembrolizumab, median (range)**	8 (1–11)	8 (1–11)	8 (1–11)	0.2
**Completed 8 or more pembrolizumab cycles**				
No	126 (33.7%)	68 (29.7%)	58 (40.0%)	0.11
Yes	241 (64.4%)	157 (68.6%)	84 (57.9%)	
Unknown	7 (1.9%)	4 (1.7%)	3 (2.1%)	
**Timing of pembrolizumab prior to surgery (months)**				
From start to surgery, median (range)	6.2 (2.2–29.7)	6.3 (2.6–29.7)	6.2 (2.2–9.4)	0.3
From end to surgery, median (range)	1.1 (0.0-26.2)	1.1 (0.0-26.2)	1.1 (0.0-7.2)	0.2
Duration of pembrolizumab, median (range)	5.1 (0.0-10.3)	5.1 (0.0-10.3)	5.1 (0–8.0)	0.3
**Cycles of NAC**,** median (range)**	8 (2, 8)	8.0 (2, 8)	8 (2, 8)	0.4
**Completed 8 NAC cycles**				
No	94 (25.1%)	49 (21.4%)	45 (31.0%)	0.05
Yes	271 (72.5%)	176 (76.9%)	95 (65.5%)	
Unknown	9 (2.4%)	4 (1.7%)	5 (3.4%)	
**Timing of NAC prior to surgery (months)**				
From start to surgery, median (range)	6.2 (2.6–29.7)	6.3 (2.6–29.7)	6.2 (2.7–9.4)	0.6
From end to surgery, median (range)	1.2 (0-26.2)	1.2 (0.4–26.2)	1.3 (0.0–6.0)	0.7
Duration of NAC, median (range)	5.0 (0.9–15.6)	5.0 (0.9–15.6)	4.9 (0.9–7.9)	0.2
**Carboplatin received**				
< 75% of doses	274 (73.3%)	168 (73.4%)	106 (73.1%)	0.8
≥ 75% of doses	92 (24.6%)	57 (24.9%)	35 (24.1%)	
Unknown	8 (2.1%)	4 (1.7%)	4 (2.8%)	
**Taxane received**				
< 75% of doses	198 (52.9%)	119 (52.0%)	79 (54.5%)	0.7
≥ 75% of doses	168 (44.9%)	106 (46.3%)	62 (42.8%)	
Unknown	8 (2.1%)	4 (1.7%)	4 (2.8%)	
**Cycles of anthracycline-cyclophosphamide**,** median (range)**	4 (0, 4)	4 (0, 4)	4 (0, 4)	0.06
**Received at least 1 cycle of anthracycline-cyclophosphamide**	324 (86.6%)	204 (89.1%)	120 (82.8%)	0.07
**Pathologic tumor classification**				
T0	232 (62.0%)	4 (2.8%)	228 (99.6%)	--
T1	94 (25.1%)	94 (64.8%)	0 (0.0%)	
T2	24 (6.4%)	24 (16.6%)	0 (0.0%)	
T3	18 (4.8%)	18 (12.4%)	0 (0.0%)	
T4	5 (1.3%)	5 (3.4%)	0 (0.0%)	
TX	1 (0.3%)	0 (0.0%)	1 (0.4%)	
**Pathologic node classification**				
N0	319 (85.3%)	228 (99.6%)	91 (62.8%)	--
N1	30 (8.0%)	0 (0.0%)	30 (20.7%)	
N2	14 (3.7%)	0 (0.0%)	14 (9.7%)	
N3	8 (2.1%)	0 (0.0%)	8 (5.5%)	
X	3 (0.8%)	1 (0.4%)	2 (1.4%)	
**Recurrence**				
No	326 (90.1%)	219 (95.6%)	107(73.8%)	< 0.0001
Yes	48 (9.9%)	10 (4.4%)	38 (26.2%)	
**Type of recurrence (for ** ***n*** ** = 48 patients with recurrence)**				
Local/regional	9 (18.8%)	0 (0.0%)	9 (23.7%)	0.09
Metastatic	39 (81.2%)	10 (100.0%)	29 (76.3%)	
**Follow-up months**,** median (95% CI)**	23.7 (22.4, 25.0)	24.1 (22.1, 25.5)	23.1 (22.2, 25.4)	0.8
**EFS (months)**,** median (95%CI)**	NR (NR, NR)	NR (NR, NR)	NR (29.4, NR)	< 0.0001
**EFS at 18 months (95% CI)**	90.7% (86.8, 93.4)	95.4% (91.0, 97.7)	83.6% (75.8, 89.0)	--
**OS (months)**,** median (95% CI)**	NR (NR, NR)	NR (NR, NR)	NR (38.5, NR)	< 0.0001

Abbreviations: NAC, neoadjuvant chemotherapy; CI, confidence interval; EFS, event-free survival; OS, overall survival; NR, not reported

Treatment discontinuations were primarily attributed to treatment-related toxicities or due to other risk factors (i.e. advanced age or comorbidities). Additional reasons for premature discontinuation of therapy included: alternative treatment schedules, insurance-related interruptions, patient preference, and suspected disease progression.

### Clinical characteristics and treatment variables according to pCR status

Of the 374 patients included, 229 (61.2%) achieved pCR (Table 1). Patients who achieved pCR were younger (*p* = 0.005) and did not have pre-existing diabetes mellitus (*p* = 0.03). Tumor grade 3 (*p* = 0.02) and a higher median Ki-67 (*p* < 0.0001) were also associated with pCR. BMI, race, ethnicity, presence of hypertension or hyperlipidemia, clinical stage, and histopathology were not significantly associated with pCR. Of the 374 patients in the cohort, BRCA status was known for 240. Of these patients, there was no difference among BRCA1/2 mutation status and pCR (12.3% in pCR vs. 9.6% in non-pCR; *p* = 0.5).

Patients who completed 8 cycles of NAC had higher rates of pCR compared to those who received less than 8 cycles of NAC (76.9% vs. 65.5%; *p* = 0.05; Table 2). The receipt of 4 cycles of anthracycline-cyclophosphamide also appeared to have higher rates of pCR, although not statistically significant (*p* = 0.06). The number of pembrolizumab cycles, carboplatin, or taxane doses received were not significantly associated with pCR. Table 3 demonstrates the number of cycles of pembrolizumab and NAC received according to pCR rate.

**Table 3:** Cycles of neoadjuvant pembrolizumab and neoadjuvant chemotherapy according to pCR rate

Neoadjuvant pembrolizumab			Neoadjuvant chemotherapy		
Number of cycles	*Number of patients*	pCR rate (95% CI)	Number of cycles	*Number of patients*	pCR rate (95% CI)
1	6	33% (0, 88)	1	--	--
2	5	40% (0, 100)	2	5	20% (0, 76)
3	7	43% (0, 92)	3	5	40% (0, 100)
4	24	75% (56, 94)	4	25	72% (53, 91)
5	27	52% (32, 72)	5	15	53% (25, 82)
6	27	41% (21, 60)	6	18	33% (9, 58)
7	30	60% (41, 79)	7	26	54% (33, 74)
8	193	68% (61, 74)	8	271	65% (59, 71)
9–11	48	54% (40, 69)	> 8	--	--

Abbreviations: pCR, pathologic complete response; CI, confidence interval

Results from univariate and multivariate logistic regression to identify predictors of pCR are shown in Table 4. A total of 367 patients were included in the multivariate analysis as 7 patients had unknown values for one or more predictors. In univariate analysis, younger age (<55 vs. 55+ years old; *p* = 0.027), absence of pre-existing diabetes (*p* = 0.034), grade 3 tumor (*p* = 0.005), and those who completed at least 8 cycles of pembrolizumab (*p* = 0.037) and 8 cycles of chemotherapy (*p* = 0.028) were associated favorably with pCR. On multivariate analysis, the lack of pre-existing diabetes (odds ratio [OR] 1.81; 95% CI, 1.06–3.11; *p* = 0.03) and grade 3 tumor (OR 2.47; 95% CI, 1.34–4.55; *p* = 0.004) remained significant. Race, ethnicity, BMI, presence of pre-existing hypertension and hyperlipidemia, stage, ER/PR status (<1% vs. ≥1%) and the receipt of any anthracycline-cyclophosphamide were not associated with pCR.

**Table 4:** Univariate and multivariate logistic regression to identify predictors of pathologic complete response

Factor	Univariate Analysis		Multivariate Analysis	
	OR (95% CI)	*P* -value	OR (95% CI)	*P*-value
Age at diagnosis (years): <55 vs. 55 or older	1.61 (1.06, 2.46)	**0.027**		
Race				
Asian vs. White	0.63 (0.33, 1.22)	0.6		
Black/African-American vs. White	0.70 (0.40, 1.22)	0.9		
Other/Unknown vs. White	0.62 (0.23, 1.66)	0.7		
Ethnicity: Hispanic vs. non-Hispanic	1.44 (0.92, 2.26)	0.11		
BMI: ≥30 vs. <30	1.26 (0.83, 1.92)	0.3		
Hypertension (no vs. yes)	1.44 (0.92, 2.23)	0.11		
Hyperlipidemia (no vs. yes)	1.12 (0.72, 1.76)	0.6		
Diabetes mellitus (no vs. yes)	1.76 (1.04, 3.00)	**0.034**	1.81 (1.06, 3.11)	**0.03**
Tumor grade: 3 vs. 2 or 1	2.42 (1.31, 4.45)	**0.005**	2.47 (1.34, 4.55)	**0.004**
Clinical stage: I or II vs. III	1.05 (0.70, 1.60)	0.8		
Estrogen receptor, %: <1% vs. ≥1%	1.01 (0.56,1.82)	1.0		
Progesterone receptor, %: <1% vs. ≥1%	1.27 (0.56, 2.88)	0.6		
Completed 8 + cycles of neoadjuvant pembrolizumab: Yes vs. No	1.59 (1.03, 2.47)	**0.037**		
Completed 8 cycles of neoadjuvant chemotherapy: Yes vs. No	1.70 (1.06, 2.74)	**0.028**		
Received at least 1 cycle of anthracycline-cyclophosphamide (AC) vs. no AC	1.78 (0.95, 3.32)	0.07		

Abbreviations: OR, odds ratio; CI, confidence interval

On further analysis of patients with and without diabetes, patients with diabetes were older (*p* < 0.001) and more likely to have obesity (*p* = 0.003), hypertension (*p* < 0.0001), hyperlipidemia (*p* < 0.0001), and advanced nodal disease (*p* = 0.002) compared to patients without diabetes (Supplementary Table 1). Patients with diabetes were less likely to complete at least 75% of taxane doses (*p* = 0.04) and received fewer cycles of anthracycline-cyclophosphamide (*p* = 0.03).

### Event-free survival and overall survival

**Fig. 1:**
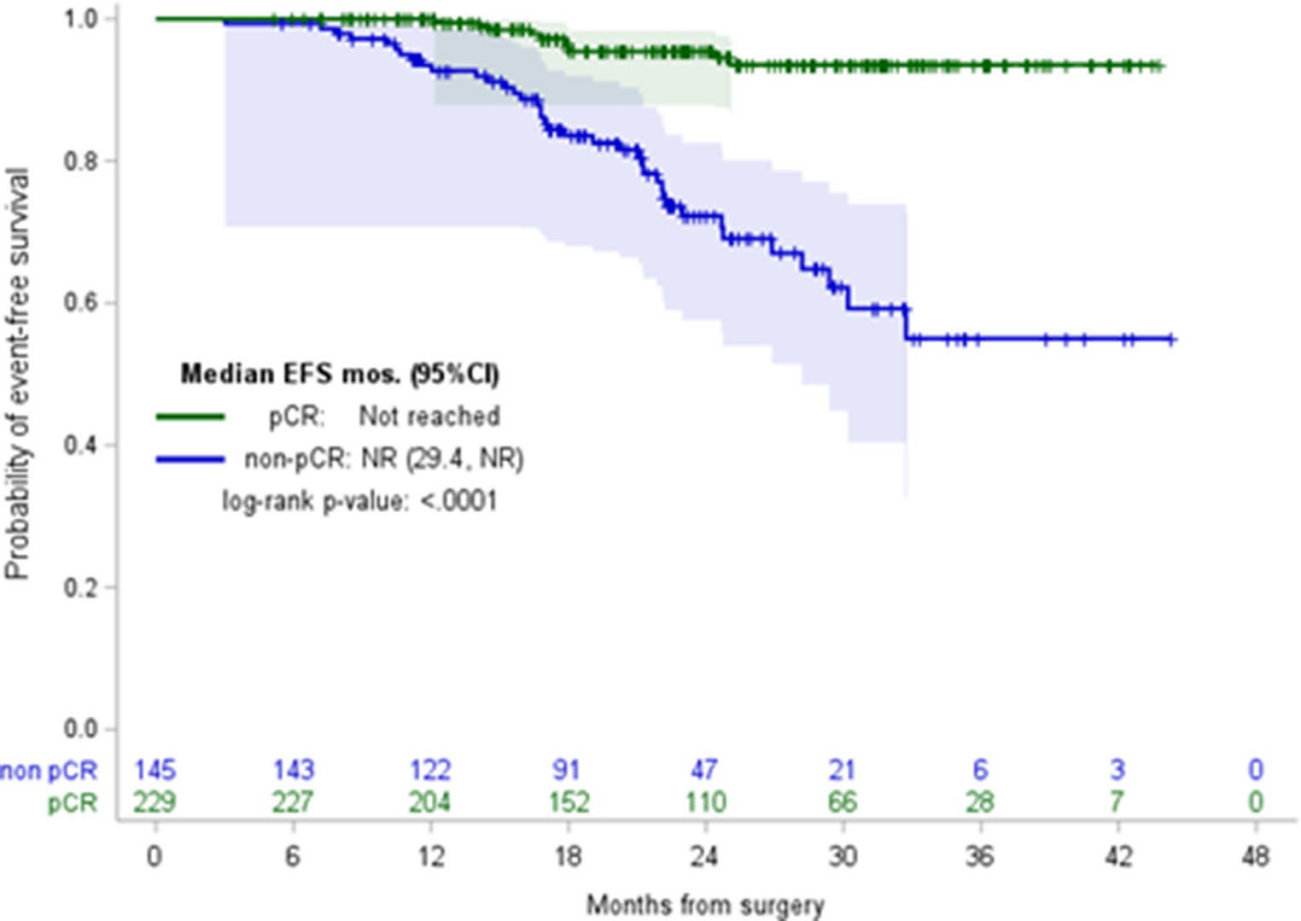
EFS by pCR status. Abbreviations: EFS, event-free survival; pCR, pathologic complete response

**Fig. 2:**
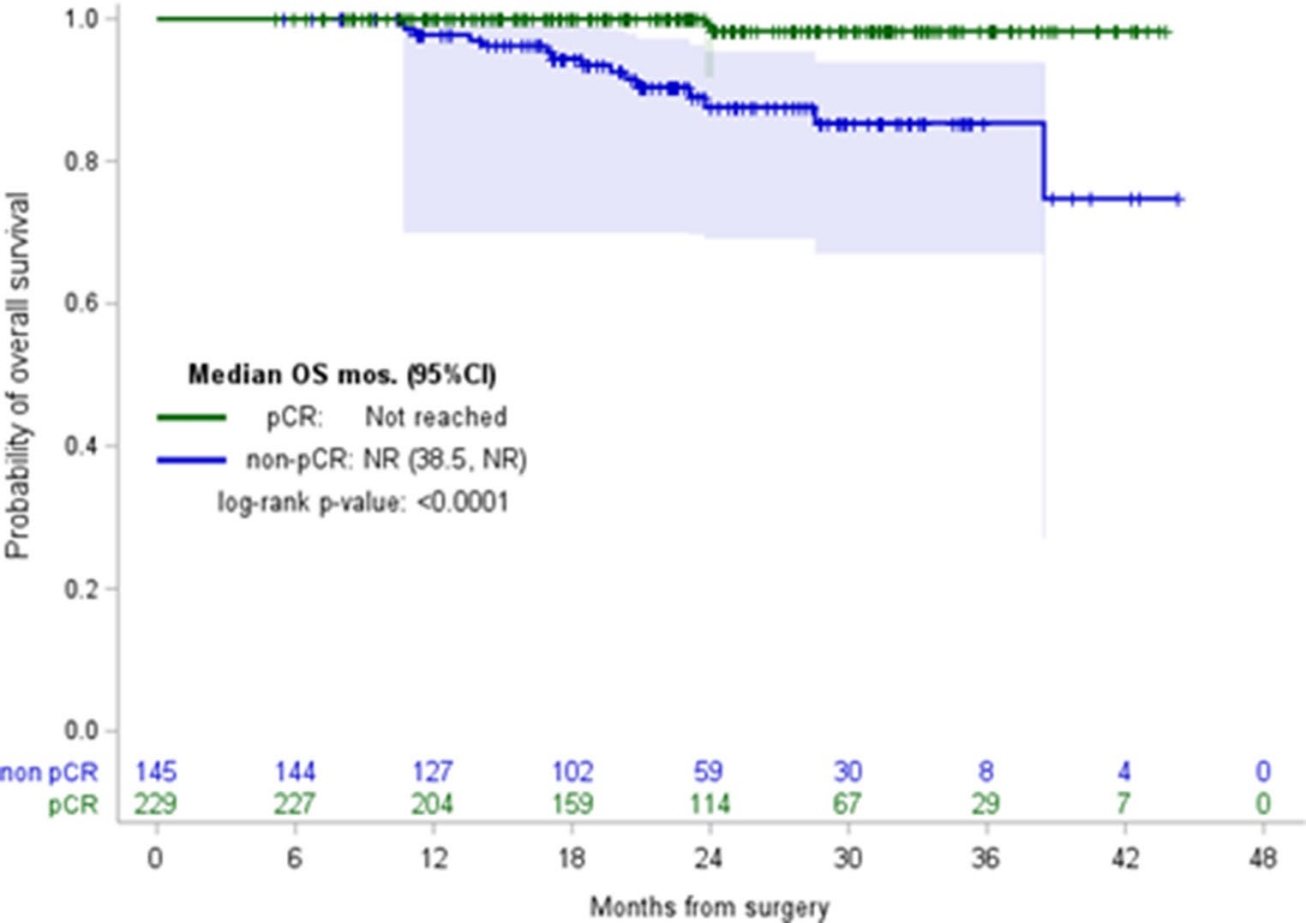
OS by pCR status. Abbreviations: OS, overall survival; pCR, pathologic complete response

With a median follow-up of 23.7 months (95% CI, 22.4–25.0) since the date of diagnosis, there were 48 recurrence events: 9 local/regional recurrences and 39 distant (Table 2). Among recurrences, 79.2% (38/48) occurred in patients with residual disease at surgery, while 20.8% (10/48) developed in those who initially achieved pCR. Estimated 18-month EFS in patients who achieved pCR was 95.4% (95% CI, 91.0-97.7) and 83.6% (95% CI, 75.8–89.0) in those with residual disease. OS was not reached in the cohort. Figures 1 and 2 illustrate EFS and OS by pCR status, respectively.

## Discussion

In this multi-institutional study of patients with early-stage TNBC treated with NAC plus pembrolizumab, 61.2% of patients achieved pCR. This approximates the 64.8% pCR rate in KEYNOTE-522 [1]. Notably, our cohort was older (median age 52 years vs. 49 years), had multiple comorbidities including diabetes mellitus in almost 20% of patients, and had a higher rate of stage III disease (52% vs. 25%) compared to KEYNOTE-522. Despite these differences, a similar pCR rate was observed, which highlights the efficacy of NAC plus pembrolizumab in a real-world cohort.

With a median follow-up of 23.7 months, EFS at 18 months in our cohort was similar (95.4%) compared to KEYNOTE-522 (91.3%), with most recurrences occurring in patients who had residual disease at the time of surgery [1]. This finding reinforces the prognostic significance of achieving pCR following NAC plus pembrolizumab in early-stage TNBC and the potential for de-escalation of therapy in the adjuvant setting for patients who achieve pCR [10]. The phase III OptimICE-pCR trial is currently ongoing to evaluate the benefit of adjuvant pembrolizumab in patients with early-stage TNBC who achieve pCR following NAC plus pembrolizumab.

In our study, patients with pre-existing diabetes had worse responses to NAC plus pembrolizumab. Diabetes can impact tumorigenesis through a number of mechanisms, including activation of the insulin and the insulin-like-growth-factor (IGF) pathway, impaired regulation of endogenous sex hormones, and metabolic inflammation [11, 12]. Diabetes can also impact the tumor microenvironment, as adipocytes adjacent to breast cancer cells exhibit cross-talk with tumor cells, which can promote tumor progression and immune modulation [12–14]. Studies show that metabolic changes such as obesity and glucose intolerance can impair T cell infiltration and function, resulting in suppressed anti-tumor immunity [15–17]. In addition to these molecular changes, patients with diabetes are more likely to experience chemotherapy-related toxicities and less likely to receive chemotherapy [18]. In our cohort, patients with diabetes were older, had a higher prevalence of other comorbidities, and received fewer cycles of taxane and anthracycline-cyclophosphamide. Further research to investigate the impact of glucose metabolism and insulin resistance in response to breast cancer therapy is needed.

The limited representation of Black patients (4.5%) in KEYNOTE-522 underscores the need for real-world validation in diverse populations. Our study demonstrated numerical trends in pCR rates by race: White patients had the highest pCR rate (64.5%), followed by Black (56.1%), Asian (53.5%), and Other/Unknown (52.9%). These patterns align with two retrospective studies that similarly reported non-significant but consistently lower pCR rates among Black patients [19, 20]. The lower pCR rates in underrepresented populations may be secondary to limited access to healthcare and being diagnosed at more advanced stages. Multiple studies also report differences in underlying tumor biology, tumor microenvironment, and immune responses among the various races, which may contribute to worse outcomes in these patients [21–29]. 

Our study also reaffirms the importance of both chemotherapy and pembrolizumab in improving clinical outcomes for patients with early-stage TNBC. We previously reported in a single-institution study of 118 patients that patients who received more cycles of pembrolizumab and NAC were more likely to achieve pCR [30]. Similarly, in this expanded study of patients, higher rates of pCR were achieved with increasing cycles of pembrolizumab and NAC received. However, in this study, patients who received 4 cycles of both NAC and pembrolizumab had the highest rates of pCR. This may reflect a selection bias and institutional differences in practice patterns amongst providers, in which interim imaging is often obtained mid-treatment and patients are recommended to proceed directly to surgery if no residual tumor is seen. Nevertheless, because of the concurrent administration of both pembrolizumab and NAC, it is difficult to tease out the optimal number of cycles of NAC and pembrolizumab to achieve pCR.

The optimal chemotherapy backbone in combination with pembrolizumab continues to be explored in clinical trials with the goal of maximizing efficacy and minimizing toxicity. In our study, more cycles of anthracycline-cyclophosphamide received trended towards higher rates of pCR, while the amount of carboplatin and taxane received were not different according to pCR status. The randomized, phase II neoPACT trial, which investigated carboplatin-docetaxel chemotherapy with pembrolizumab for 6 cycles in patients with stage I-III TNBC, reported a pCR rate of 58%, suggesting that an anthracycline-free chemo-immunotherapy regimen may be possible without compromising efficacy for patients unable to tolerate an anthracycline [31]. The phase III SCARLET trial (NCT05929768) is comparing the neoPACT regimen to KEYNOTE-522 and will hopefully shed some light on the efficacy of an anthracycline-free regimen.

Similar to the neoPACT trial, patients with tumors of low ER and PR expression (as defined by IHC 1–10%) were included in the present study given the similar molecular characteristics and clinical outcomes to TNBC [32–35]. In this study, patients with low ER/PR (1–10%) tumors had similar pCR rates to those with tumors of ER/PR <1%. This observed pattern supports the potential eligibility of patients with low ER/PR (1–10%) expression for consideration of immunotherapy, as suggested by the European Society for Medical Oncology (ESMO) Clinical Practice Guidelines [36]. 

Limitations of this study include being retrospective in nature which precluded the ability to compare the KEYNOTE-522 regimen with a control cohort. Our short follow-up time also limited the ability to identify predictors of EFS and OS. Additionally, chemotherapy dose reductions were not included which can affect treatment response, and details regarding type of adverse events were not addressed.

In conclusion, this multi-institutional study of patients with early-stage TNBC treated with NAC plus pembrolizumab supports the effectiveness of KEYNOTE-522 in a diverse, real-world population. Patients with diabetes mellitus and low tumor grade were less likely to achieve pCR. This study highlights the importance of baseline health status in influencing the response to breast cancer therapy and raises the possible impact of glucose metabolism on chemo-immunotherapy resistance.

## Data Availability

Data available on request from the authors.
